# Comparative Effects of Turmeric Secondary Metabolites Across Resorptive Bone Diseases

**DOI:** 10.3390/metabo15040266

**Published:** 2025-04-11

**Authors:** Laura E. Wright, Jennifer B. Frye, Andrew G. Kunihiro, Barbara N. Timmermann, Janet L. Funk

**Affiliations:** 1 College of Medicine, Ohio State University, Columbus, OH 43210, USA; laura.wright@osumc.edu; 2 Department of Medicine and School of Nutritional Sciences and Wellness, The University of Arizona, Tucson, AZ 85724, USA; jabeisch@arizona.edu (J.B.F.);; 3 Department of Medicinal Chemistry, The University of Kansas, Lawrence, KS 66045, USA; btimmer@ku.edu

**Keywords:** turmeric, curcuminoids, curcumin, essential oils, polysaccharides, bone, arthritis, osteoporosis, breast cancer, bone metastases

## Abstract

**Background**: Turmeric (*Curcuma longa* L.) rhizomes, whose secondary metabolites include polyphenols and terpenoids, have been used medicinally for millennia. However, modern scientific inquiry has primarily focused on medicinal effects of turmeric’s polyphenolic curcuminoids, including when evaluating turmeric use to maintain bone health. **Methods**: Disease-specific biological effects of turmeric’s major secondary metabolites (polyphenols and/or terpenoids), with or without associated turmeric rhizome-derived polysaccharides, were determined in vivo using pre-clinical models of clinically relevant resorptive bone diseases induced by different mechanisms. These included inflammatory arthritis, cancer-driven osteolytic bone metastases, and hormone deficiency-driven post-menopausal osteoporosis. **Results**: In the arthritis model, the safety profile of curcuminoids alone was superior. However, curcuminoids and terpenoids each had anti-inflammatory effects and prevented bone resorption, with polysaccharide-containing curcuminoid extracts having greater effect than curcuminoids alone. In the human osteolytic breast cancer bone metastases model, curcuminoid extracts containing polysaccharides tended to yield greater effects in reducing bone osteolysis and tumor progression than curcuminoids alone or more complex extracts. In contrast, only purified curcuminoids prevented bone loss in a post-menopausal osteoporosis model, while polysaccharide-containing curcuminoid extracts were without effect. In vitro metabolite effects on disease-specific mechanistic pathways in synoviocytes, osteoclasts, or breast cancer cells were consistent with documented in vivo outcomes and included differential metabolite-specific effects. **Conclusions**: In summary, these findings suggest that turmeric’s potential medicinal musculoskeletal effects are complex, pathway- and target-specific, and not limited to curcuminoids, with safety concerns potentially limiting certain uses.

## 1. Introduction

Traditional ethnobotanical uses of certain plants for medicinal purposes still influence their contemporary applications and scientific study today. Turmeric (*Curcuma longa* L.) is one such botanical. While crude preparations of turmeric rhizome have been used for millennia in Ayurvedic medicine for their anti-inflammatory properties [1], modern use has primarily focused on turmeric’s polyphenols (curcuminoids), while largely excluding consideration of medicinal benefits of turmeric’s terpene-enriched essential oils [2,3,4,5]. Potential isolated or combined medicinal benefits of these two classes of secondary metabolites—either with or without other rhizome components, such as polysaccharides—remain underexplored in scientific research and commercial formulations, particularly regarding their potential additive or interactive effects. For example, while turmeric has been one of the top-selling botanical dietary supplements in the United States for over a decade, almost all commercially available products sold are specifically formulated to only contain curcuminoid-enriched (curcuminoid-only) fractions extracted from the rhizome [6]. More specifically, most products contain a mixture of structurally related curcuminoids (≥95% curcuminoids), comprised of curcumin, demethoxycurcumin, and bis-demethoxycurcumin, which are chemically difficult to isolate and may have distinct biological effects [7], with evidence that less costly synthetic curcumin, rather than turmeric rhizome-derived curcuminoids, may be incorporated into some commercial turmeric dietary supplements [6,8,9].

Polyphenolic curcuminoids and related diarylheptanoids, which are responsible for turmeric’s orange color and are a focus of medicinal interest, comprise approximately 3–5% by weight of dried ground turmeric rhizomes [3]. Turmeric’s volatile essential oils (TEOs), comprising 4% by weight of the rhizome, are a complex mixture of more than 80 hydrophobic terpenoids, including mono-, di-, and sesquiterpenes, whose major components include ar-turmerone, α-turmerone, and β-turmerone [10,11,12]. Medicinal effects of TEO, while much less frequently studied than curcuminoids, have been reported and include properties similar to those reported for curcuminoids, including anti-inflammatory, anti-cancer, and antioxidant effects [3,11,13,14,15]. In addition to these two distinct classes of turmeric secondary metabolites, turmeric rhizome-derived polysaccharides (2% yield by weight with aqueous extraction) have also been reported to have anti-inflammatory and antioxidant properties [16,17].

Botanical dietary supplements, including turmeric supplements, are widely used in the United States to promote musculoskeletal health [18,19]. In populations with a high burden of bone disease, use of turmeric dietary supplements to promote bone health is particularly prevalent, including individuals with rheumatoid arthritis (30%) and post-menopausal breast cancer survivors (23%) [20]. However, clinical interest in turmeric for musculoskeletal disorders has primarily concentrated on curcuminoids [2,5,21], with musculoskeletal disorders representing 17% of curcuminoid-related clinical trials, second only to studies focused on metabolic dysfunction (28%) [4].

To fill this knowledge gap, pre-clinical studies evaluating medicinal bone effects of turmeric-derived secondary metabolites, in isolation or combination, with or without polysaccharides, were undertaken. The in vivo effects of a crude turmeric extract vs. secondary metabolite-enriched fractions (e.g., curcuminoids-only, with or without polysaccharides, versus turmeric essential oil [TEO]-only) were determined and compared in head-to-head pre-clinical studies modeling three different bone disorders that share a common clinical manifestation (bone resorption), but, importantly, are driven by different mechanisms. Specifically, the effects of complex turmeric extracts and/or fractions were compared in pre-clinical models of (1) rheumatoid arthritis, an inflammatory disease, where tumor-like growth of the inflamed joint synovium drives adjacent bone destruction [22]; (2) osteolytic breast cancer bone metastases, where tumor-adjacent bone resorption is tumor driven [23]; and (3) ovariectomized rats, a Federal Drug Administration (FDA)-mandated model in the United States for testing therapeutics targeting post-menopausal bone loss, which is driven by systemic changes in reproductive hormones [24].

## 2. Materials and Methods

### 2.1. Turmeric Extract Preparation and Analyses

All turmeric products studied here met the rigorous Natural Product Integrity guidelines set forth by the United States National Institutes of Health (NIH) National Center for Complementary and Integrative Health [25], which reviewed and approved all turmeric products described here. Dried turmeric rhizome powder or a commercial curcuminoid-only product were purchased from San Francisco Herb and Natural Food (San Francisco, CA, USA) or Fisher Scientific (ACROS Organics, Pittsburgh, PA, USA, “curcumin 98+%”, no. 218580100), respectively. High-performance liquid chromatography (HPLC) analyses guided fractionations and documented metabolite content (e.g., curcuminoids vs. terpenes) of each preparation prior to use, with detailed extraction and analytic methods as previously described [11,26]. The extract polyphenol content is reported as total curcuminoids by weight, comprised of curcumin (64–79% of total curcuminoids), demethoxycurcumin (16–21%), and bis-demethoxycurcumin (5–15%). In brief, methanol extraction of the dried rhizome was used to prepare a crude turmeric extract (9.5% yield) containing equal parts curcuminoids (34%), essential oils (~33% sesquiterpenoids), and polysaccharides (~33%). A turmeric essential oil (TEO) fraction, devoid of curcuminoids or polysaccharides, was prepared by hexane extraction of the dried rhizome (3.7% yield). A TEO-free curcuminoid-containing fraction (41%) that also contained polysaccharides (~59% polar compounds) (CURC/POLAR extract) was prepared by methanol extraction of the hexane-marc (3.1% yield). A commercial curcuminoids-only extract contained 94% curcuminoids by HPLC, despite commercial description as 98+% curcumin (Fisher Scientific, Pittsburgh, PA, USA). For head-to-head comparisons of turmeric preparations, in vivo treatments were normalized to curcuminoid dose (mg/kg) or an equivalent by dose by weight of TEO. For all experiments described here, extracts (vs. vehicle alone [dimethyl sulfoxide]) were administered via intraperitoneal (IP) dosing route to eliminate possible matrix effects altering oral metabolite absorption (e.g., effects of TEO and/or polysaccharides on curcuminoid bioavailability), as has been described [27,28,29,30,31,32].

### 2.2. Animal Procedures

All animal procedures were conducted under Institutional Animal Care and Use Committee (IACUC) approved protocols, consistent with American Association for Laboratory Animal Science guidelines [33]. Sample size (n = 4, 8, 10, or 12 for control, arthritis, bone metastases, or osteoporosis models) was based on power analyses using prior primary endpoint data for each model (joint inflammation, bone metastases size, and trabecular bone loss, respectively), assuming a moderate effect size with α = 0.05 and β = 0.8 (G*Power software v3.1) [34]. Animals were randomly allocated to groups, with order of manipulations (e.g., tumor-cell injection) or treatments also randomized to minimize confounder effects. Observers were blinded to treatment groups when assessing primary endpoints. All animals were included for analyses, except for those eliminated for technical reasons (e.g., anesthesia-related deaths or issues with intracardiac injections). For some endpoints, experimental results from replicated experiments were combined.

For the rheumatoid arthritis model (Table 1), female Lewis rats (Inotiv, Indianapolis, IN, USA) were administered peptidoglycan–polysaccharides (25 g rhamnose/gm body weight) isolated from the sonicated cell wall of group A Streptococcus pyogenes (SCW, Lee Laboratories, Grayson, GA, USA), via intraperitoneal (IP) injection (vs. vehicle [controls]) to induce joint inflammation [11,26,35]. Treatments (IP) with indicated turmeric preparations (or vehicle alone) were begun four days prior to SCW inoculation and continued daily for 8 days, then 5 days a week, until end of experiment (28 days post SCW inoculation). Data are reported for rats treated with turmeric preparation dosed as indicated (curcuminoids: 4, 23 or 48 mg/kg/d [human equivalent dose {HED} ≈ 40, 225, 465 mg/d [36]] or TEO: 28 or 56 mg/kg/d [HED ≈ 270 or 540 mg/d]). Joint inflammation attributable to SCW joint deposition, which closely mimics the pathology of human rheumatoid arthritis [35,37], was scored daily in each distal limb in a blinded manner as previously described and is reported here for day 28 (experiment endpoint). Treatment effects on histologic granuloma formation, a host response to hepatic SCW deposition, are also reported [35].

For the osteolytic bone metastases model (Table 1), four-week-old female athymic nude mice (Inotiv, Lafayette, Indiana, USA) were inoculated in the left cardiac ventricle with human MDA-MB-231 breast cancer cells (1 × 10^5^ cells), as previously described [38,39]. In brief, beginning the day after tumor inoculation, mice were treated daily five days per week (IP) with the turmeric extracts indicated (normalized to 50 mg curcuminoids/kg/d [HED = 245 mg/d]) vs. vehicle alone. Mice were imaged radiographically (Digital Faxitron MS-20, Tucson, AZ, USA) at indicated times (weekly) to document osteolytic bone metastases formation, with osteolytic lesion size analyzed by three blinded observers using ImageJ 1.43u software (National Institutes of Health, Bethesda, MD, USA). In separate experiments, curcumin vs. curcumin glucuronide levels were assayed within bone following intraperitoneal (IP) or oral treatment of mice with 100 or 500 mg/kg CURC, respectively, with β-glucuronidase enzyme activity determined within bone in separate sets of mice, comparing across mouse strains for β-glucuronidase (GUSB)-wild type (C57Bl/6), GUSB-low (C3H/HeJ), and GUSB-null (C67Bl/6, mps/msp) mice [40].

For the rat ovariectomized (OVX) model (Table 1) of post-menopausal osteoporosis [24,41], pair-fed, 3-month-old female Sprague–Dawley rats (Inotiv) were dosed IP daily beginning on the day of surgery (OVX or sham) for two months with the indicated doses of turmeric extracts (vs. vehicle alone), which were normalized to the curcuminoid content (60 mg/kg/d, [HED 580 mg/d]).

As indicated, additional in vivo study endpoints included determination of circulating white blood cell counts and hematocrits using a Hemavet 880 analyzer (CDC Technologies, Oxford, CT, USA) or serum alanine aminotransferase (ALT) levels using an Endocheck Plus Chemistry Analyzer (Hemagen Diagnostics, Columbia, MD, USA). C-reactive protein levels in serum collected at end of experiment and stored at −80 °C were assayed without prior freeze thaw using a commercial ELISA (catalog #557825, BD Biosciences, Franklin Lakes, NJ, USA). Bone mineral density (BMD) of the distal 25% of excised hind limb femurs for rat OVX or SCW models are reported from end of experiment using a Piximus densitometer (GE Lunar, Madison, WI, USA). Resorptive damage to bone microarchitecture was documented in all models by micro-computerized tomography (microCT) (VivaCT 40, Scanco, Basserdorf, Switzerland) using standard procedures.

### 2.3. Cell Culture Methods

Effects of turmeric extracts on human vascular endothelial cells were determined using pooled human umbilical vein endothelial cells (HUVECs) purchased from Cascade Biologics and treated, as previously described, with turmeric extracts beginning 1 h prior to stimulation with tumor necrosis factor-alpha (TNFα 10 ng/mL) or media alone [42]. After 4 h of TNFα treatment, mRNA was harvested (n = 3 condition) for reverse transcription polymerase chain (RT-PCR) analysis using standard methods. Human specific primers for intercellular adhesion molecule-1 (ICAM-1) and an 18S primer (as an internal control; Hs99999901_s1) obtained from Applied Biosystems (Foster City, CA, USA) were used to determine gene expression with data analyzed using the comparative C_T_ method as means of relative quantitation of gene expression, normalized to the endogenous reference (18S RNA) and relative to a calibrator, as described by the manufacturer (Applied Biosystems) [42]. Additionally, for analysis of turmeric extract effects on endothelial cell NF-κB activation, nuclear protein was isolated from HUVECs treated for 4 h with turmeric extracts prior to 1 h of stimulation with TNFα (5 mg/mL) to determine nuclear p65 NF-κB protein levels by Western analysis using a primary antibody directed against the p65 NF-κB protein (Cell Signaling Technology, Danvers, MA, USA).

Human synovial fibroblasts previously isolated from subjects undergoing joint replacement surgery who were diagnosed with rheumatoid arthritis, as defined by the American College of Rheumatology, were treated concurrently with interleukin-1 beta (IL-1β, 30 ng/mL) and the indicated turmeric extract doses for 24 h. The parathyroid hormone-related protein (PTHrP) or prostaglandin E2 (PGE_2_) content of the conditioned media was assayed using a commercial radioimmunoassay (RIA, Nichols Institute, San Juan Capistrano, CA, USA) or enzyme-linked immunosorbent assay (ELISA, R&D Systems, Minneapolis, MN, USA), respectively [43]. Cell viability was unchanged by treatments, as determined by 3-(4,5-dimethylthiazol-2-yl)-2,5-diphenyltetrazolium bromide (MTT) assay (Promega, Madison, WI, USA). Transforming growth factor-beta (TGFβ, 5 ng/mL)-stimulated PTHrP secretion was also assayed in protease-treated, conditioned media of human breast cancer MDA-MB-231 cells using a commercial immunoradiometric PTHrP assay (#DSL-8100, Beckman Coulter, Indianapolis, IN, USA) [38].

Turmeric extract effects on osteoclast (Oc) formation were determined using primary rat bone marrow monocytes obtained from the femora of 3-month-old female Sprague–Dawley rats under aseptic conditions, which were treated with receptor activator of nuclear factor-κB ligand (RANKL, 50 ng/mL; R&D Systems) and macrophage colony-stimulating factor (M-CSF, 50 ng/mL; R&D Systems) to stimulate osteoclastogenesis and co-treated with indicated turmeric extract concentrations (or vehicle). Cells were fixed on d 5 prior to staining for tartrate-resistant acid phosphatase (TRAP; Sigma-Aldrich, Burlington, MA, USA) activity to visualize osteoclast cells [43]. TRAP+ multinucleated (>3) cells were counted in each well, with data expressed as total number of osteoclasts per well (mean ± SEM), or as % of control. In osteoclastogenesis studies using a murine macrophage osteoclast precursor (RAW 264.7), cells were pre-incubated with botanical treatments for 4 h prior to RANK-L (50 ng/mL) treatment to stimulate osteoclastogenesis [44], with TRAP+ multinucleated OC counted 5 days post-start of RANKL treatment, as described above. To determine turmeric effects on NF-κB activation in Oc precursors, nuclear protein was extracted after one hour of RAW 264.7 cell RANKL treatment using a commercial kit (Panomics, Fremont, CA, USA) and assayed for nuclear factor kappa B (NF-κB) p50 binding activity using a commercially available ELISA (#70-515, Millipore, Temecula, CA, USA).

### 2.4. Statistical Analyses

All statistical analyses were performed using Prism 10 software (GraphPad, San Diego, CA, USA), with values presented as mean ± SEM. Statistical significance was determined as appropriate using one- or two-way analysis of variance (ANOVA) with post-hoc testing or other indicated analyses (e.g., linear regression). Data presented in radar plots depict statistically significant effects of treatments in blocking arthritis-induced effects, as determined by one-way ANOVA across treatment groups (control, turmeric, arthritis, and arthritis + turmeric).

## 3. Results

### 3.1. Characterization of Turmeric Extracts for In Vivo Testing in Bone Resorption Models

**Figure 1:**
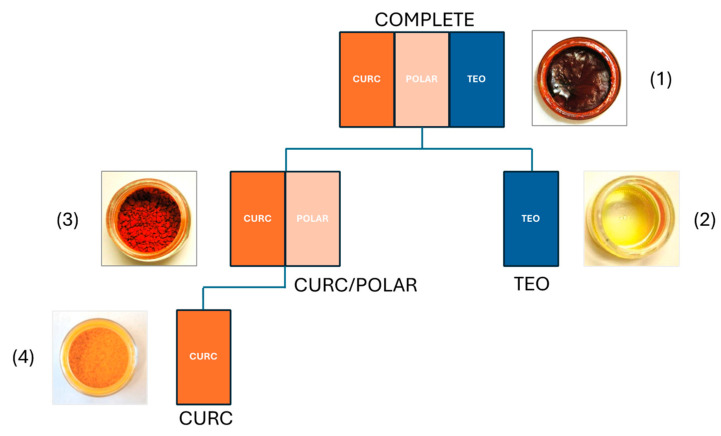
Isolation scheme for turmeric extracts tested in vivo. A (1) crude turmeric extract (COMPLETE) containing approximately equal parts curcuminoids (34% by HPLC), terpene-enriched essential oils, or polar components, including polysaccharides, was isolated by methanol extraction of dried turmeric rhizomes. A (2) turmeric essential oil (TEO) fraction, and (3) TEO-free fraction containing curcuminoids (41% by HPLC) and polar compounds (CURC/POLAR) were isolated by hexane extraction or methanol extraction of the hexane marc, respectively. Lastly, (4) a “98%” curcuminoids-only (94% by HPLC) commercial product was purchased.

**Figure 2:**
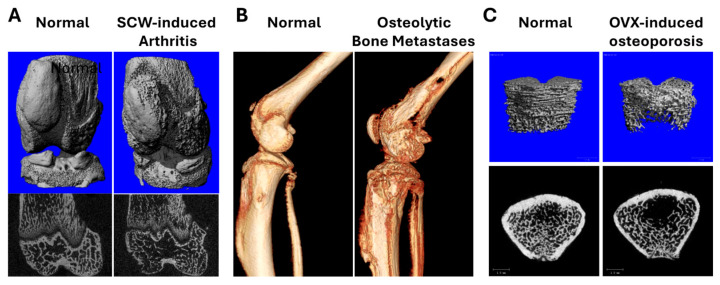
Representative three-dimensional or cross sectional micro-CT images of hind limb bone loss (vs. controls) from each of the three bone resorption models, including (**A**) periarticular loss of trabecular bone and cortical bone damage in rats with inflammatory SCW-induced arthritis, which mimics the inflammatory joint pathology of rheumatoid arthritis; (**B**) focal osteolytic hind limb bone destruction associated with progression of bone-metastatic human breast cancer cells in a mouse model; and (**C**) trabecular bone loss in ovariectomized (OVX) rats, a model of post-menopausal osteoporosis.

Experimentally prepared turmeric extracts isolated from dried turmeric rhizomes were characterized for turmeric metabolite content and stability prior to use in bone resorption models (Figure 1). Dosing of turmeric preparations across models was normalized to curcuminoid content (or an equivalent dose by weight of TEO), comparing: (1) a crude (“COMPLETE”) extract containing both secondary metabolites (curcuminoids 33.7% by weight; TEO, ~33%) and polar (e.g., polysaccharide) materials (~33%); (2) a TEO-only fraction (“TEO”); (3) an extract, devoid of TEO, containing only curcuminoids (40.6%) and polar materials (“CURC/POLAR”); or (4) a curcuminoid enriched (94%) product (“CURC”) devoid of other metabolites. Extracts, normalized to metabolite content, were tested for effects on bone resorption in three pre-clinical bone disease models where osteoclast-mediated resorption of trabecular bone, driven by different mechanisms, is a hallmark, including (Figure 2) (1) rheumatoid arthritis (Figure 2A); (2) osteolytic breast cancer bone metastases (Figure 2B); and (3) post-menopausal osteoporosis (Figure 2C). Given evidence that TEO and/or polysaccharides can significantly alter CURC oral bioavailability [27,28,29,30,31,32], all extracts were normalized to metabolite dose and administered IP for comparison of pharmacodynamic effects of normalized turmeric metabolites doses across models and across extracts of varying complexity.

### 3.2. Turmeric Metabolite Effects in an Inflammatory Rheumatoid Arthritis Model

**Figure 3:**
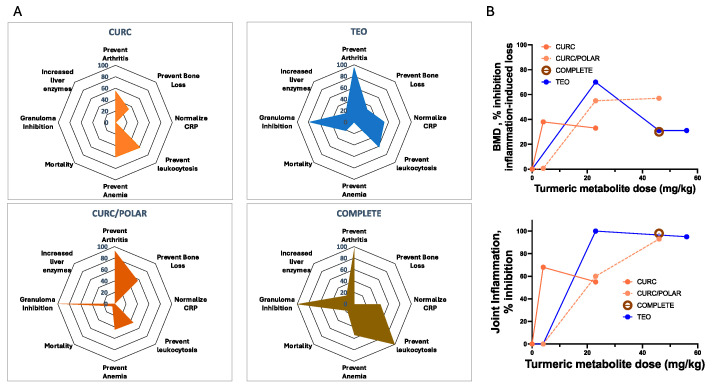
Turmeric treatment effects in SCW-induced arthritis. (**A**) Radar plots summarize statistically significant effects (*p* < 0.05 by one-way ANOVA) of each turmeric extract in the SCW-model. Data are expressed as % inhibition of SCW-induced changes (vs. controls), or in the case of abnormal liver function or mortality, % prevalence with n = 8–53/group. Adverse effects are listed to the left and beneficial effects to the right of each plot. Results graphed represent the highest extract dose tested, where anti-arthritic effects plateaued for each extract (CURC 23 mg/kg; CURC/POLAR 48 mg/kg curcuminoids; TEO 56 mg/kg; COMPLETE 48 mg/kg curcuminoids). (**B**) Dose-dependent turmeric extract effects on arthritis-induced loss of proximal femoral bone mineral density (BMD) (upper panel) are compared to dose dependent effects on joint inflammation (lower panel). Turmeric extract doses are as indicated, with only a single dose of COMPLETE extract tested (48 mg/kg/d).

Anti-inflammatory effects of each of the four turmeric extract in a rheumatoid arthritis model, some of which have been reported previously for isolated extracts [11,26,43], are compared here in radar plots summarizing statistically significant outcomes, with clinically beneficial effects to the right and adverse effects on the left (Figure 3A). While most commercial turmeric products sold in the United States only contain purified curcuminoids (CURC) [6], this extract was the least effective in blocking joint inflammation, as compared to the more chemically complex curcuminoid-containing extracts (CURC/POLAR, COMPLETE) or TEO alone. Notably, each of turmeric’s secondary metabolites had significant anti-arthritic effects, with the benefits of TEO-only extracts exceeding that of CURC-only. However, TEO-only extracts and more complex curcuminoid-containing extracts (COMPLETE or CURC/POLAR) had less favorable safety profiles (vs. CURC only), including increased mortality, prevalence of abnormal liver function and/or inhibition of granuloma formation, a protective host response for certain chronic infectious diseases (e.g., tuberculosis), where quiescent infections can be reactivated when granuloma formation is suppressed [45]. Key to these studies, turmeric extract effects on periarticular bone loss (Figure 3B, top panel [distal femoral BMD]) were dose- and extract-dependent, with each extract demonstrating statistically significant, beneficial effects in preventing inflammation-driven bone resorption. Notably, effects of each turmeric metabolite or extract on bone destruction in the RA model closely mirrored their effects in reducing joint inflammation (Figure 3B, bottom panel).

Certain treatment responses in the RA model were metabolite-specific (Figure 3A). For example, polysaccharide-containing extracts had the most pronounced (adverse) effect on granuloma formation, while only curcuminoid-containing extracts prevented disease-related anemia, with increased efficacy in preventing leukocytosis. In contrast, TEO-containing extracts were notable for their unique ability to reduce circulating C-reactive protein (CRP) levels, which are used clinically to monitor RA disease activity [46]. The reason for the lack of correlation for a given extract between anti-inflammatory effects on joints and levels of CRP, an acute phase reactant also associated with cardiovascular disease risk [46,47], is unclear.

**Figure 4:**
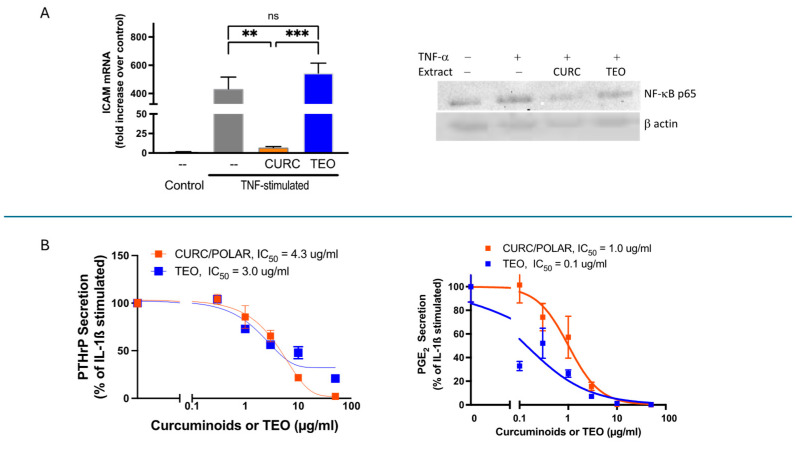
Effects of CURC vs. TEO on human vascular endothelial or synovial cells. (**A**) TNFα-induced ICAM gene expression in human umbilical vein endothelial cells (HUVEC), as determined by RT-PCR (n = 3/group) (left panel), was inhibited by 1 h pre-treatment with CURC (25 ug/mL) but not TEO (25 ug/mL). TNFα-induced activation of NF-κB in HUVECs, as determined by Western analysis of nuclear NF-κB p65 protein levels (right panel), was also inhibited by 4 h of pre-treatment with CURC (10 ug/mL) but not TEO (10 ug/mL). ** *p* < 0.01, vs. TNFα-stimulated; *** *p* < 0.001, CURC vs. TEO. ns = not significant. (**B**) In primary cultures of human synoviocytes isolated from rheumatoid arthritis joints treated concurrently with IL-1β and the indicated doses of CURC or TEO, secretion of bone-destroying PTHrP (left panel) or PGE_2_ (right panel) were both significantly inhibited in a dose dependent fashion by each of turmeric’s secondary metabolites.

TEO vs. CURC effects on RA-relevant human cell lines in vitro were examined to expand translational significance, revealing additional target-specific differential effects of these two secondary metabolites (Figure 4). Because of the high burden of vascular inflammation and associated cardiovascular disease (CVD) in RA, effects of CURC vs. TEO on tumor necrosis factor-alpha (TNFα)-driven activation of human vascular endothelial cells were explored (Figure 4A). In contrast to TEO-only blockade of CRP, CURC (but not TEO) blocked TNFα-stimulated activation of human endothelial cells (e.g., increased ICAM in HUVECs) (Figure 4A, left panel), an effect likely mediated by CURC-specific inhibition of nuclear factor kappa B (NF-kB) activation (Figure 4A, right panel).

Because inflammatory mediators secreted from the tumor-like synovium in RA joints drive bone destruction in this disease, effects of CURC vs. TEO on human RA synoviocyte IL-1β-stimulated secretion of the bone-destroying parathyroid hormone-related protein (PTHrP) or prostagalandin-E_2_ (PGE_2_) were also examined [35,48,49,50]. Consistent with their isolated in vivo anti-inflammatory joint effects, each metabolite (curcuminoids or TEO) also blocked secretion of both bone destruction mediators in a dose-dependent fashion with a similar IC_50_ for PTHrP (Figure 4B, left panel) and greater TEO potency for PGE_2_ inhibition (Figure 4B, right panel).

**Figure 5:**
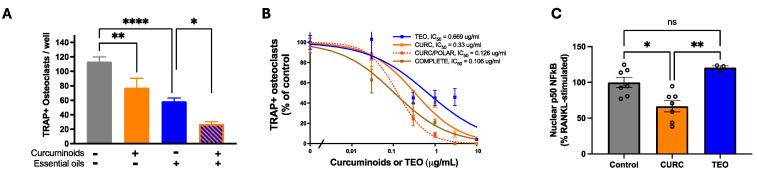
Direct turmeric metabolite effects on osteoclast formation. (**A**) Effects of CURC vs. TEO (4 h pre-treatment) on RANK-L (50 ng/mL)-induced osteoclast formation from RAW 264.7 macrophage cells with results expressed as the number of multinuclear TRAP+ osteoclasts formed per well (n = 4–5 per treatment). * *p* < 0.01 TEO vs. TEO + CURC; ** *p* < 0.01 CURC vs. control; **** *p* < 0.0001 TEO vs. control. (**B**) Dose-dependent turmeric extract effects on RANKL-stimulated multinuclear TRAP+ osteoclast formation in primary bone marrow cultures (n = 4–5/treatment expressed as % of untreated control). (**C**) Effect of CURC vs. TEO (4 h pre-treatment) on nuclear p50 NF-κB protein levels, as determined by ELISA, one hour after RANK-L (50 ng/mL) stimulation of RAW 264.7 cells (n = 3–7/group) vs. RANKL-only treated controls, where nuclear p50 NF-κB levels increased 2.7-fold (*p* < 0.01). * *p* < 0.05 CURC vs. control. ** *p* < 0.01 CURC vs. TEO. ns = not significant.

Effects of both turmeric metabolites on RANKL-driven, bone-resorbing osteoclast (Oc) formation, a final cellular pathway driving bone loss in all resorptive bone diseases [51,52], were examined in vitro. Each secondary metabolite blocked RANKL-stimulated Oc formation from Oc precursors (Figure 5A), with additive effects in combination. Similar effects on Oc formation were noted when RANKL-stimulated primary bone cultures were treated (Figure 5B), with the COMPLETE extract yielding the most potent effects (lower IC_50_). Because NF-κB signaling is one pathway driving RANKL-stimulated Oc formation [52], metabolite effects of RANKL-stimulated NF-κB activation in Oc precursors were also examined. Consistent with HUVEC findings, CURC, but not TEO, reduced NF-κB activation in Oc precursors in response to RANKL stimulation (Figure 5C), suggesting blockade of osteoclast formation by TEO occurs via a different mechanism.

### 3.3. Turmeric Metabolite Effects in an Osteolytic Breast Cancer Bone Metastases Model

**Figure 6:**
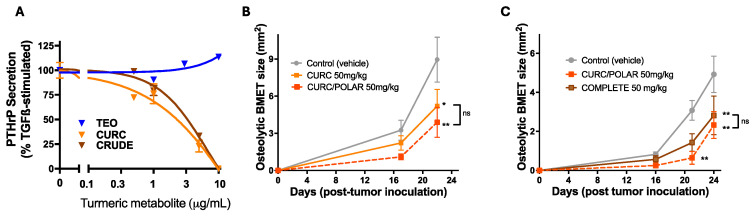
Effects of turmeric metabolites on bone-tropic human breast cancer cells in vitro and in vivo. (**A**) Human MDA-MB-231 cells (n = 4–8/group) were pre-treated in vitro with the indicated doses of turmeric extracts for 4 h prior to stimulation with TGFβ (5 ng/mL) for 24 h with conditioned media then assayed for the PTHrP content (n = 4–8/group). Half maximal inhibitory concentrations were determined using a four-parameter logistic equation. (**B**) Effects of CURC vs. CURC/POLAR extracts (50 mg/kg curcuminoids) on osteolytic breast cancer bone metastases formation was determined radiographically in female nude mice inoculated with MDA-MB-231 cells (vs. vehicle-treated controls) when mice were treated with turmeric extracts five days per week until the end of experiment. (**C**) Effects of CURC/POLAR vs. COMPLETE extract on osteolytic breast cancer bone metastases formation was similarly determined in a separate similarly designed experiment. Difference between treatments were determined by two-way ANOVA (n = 10/group). * *p* < 0.05; ** *p* < 0.01. ns = not significant.

Focal bone loss driven by the inflammatory tumor-like synovium in RA and tumor-driven osteolysis in bone-metastatic breast cancer share some common characteristics, including a key role for parathyroid hormone-related protein (PTHrP) secretion from tumor-(like) masses in both processes, which drives RANKL production, and thus osteocyte (Oc) formation and focal bone resorption [49,53]. Because CURC and TEO inhibited IL-1β-stimulated PTHrP from human rheumatoid synoviocytes, secondary metabolite effects on PTHrP secretion from human breast cancer cells were examined. Interestingly, only curcuminoid-containing extracts (Figure 6A) inhibited TGFβ-stimulated PTHrP secretion from human breast cancer cells that form TGFβ-dependent, PTHrP-driven osteolytic bone metastases (BMET) in vivo [38]. However, given the evidence that CURC and TEO each blocked RANKL-stimulated osteoclast formation (Figure 5), which occurs downstream of PTHrP, the comparative in vivo effects of curcuminoid-containing extracts of varying complexity on osteolytic BMET formation in the breast cancer model were examined. Interestingly, curcuminoid-containing extracts of any complexity (Figure 6B, CURC vs. CURC/POLAR; Figure 6C, CURC/POLAR vs. COMPLETE) significantly reduced progression of osteolytic breast cancer lesions. While differences in head-to-head experiments between extracts were not significantly different, increased extract complexity tended to track with greater treatment benefits, with curcuminoid-only extracts containing polysaccharides (CURC/POLAR) tending to have the greatest inhibitory effect (Figure 6B,C).

### 3.4. Turmeric Metabolite Effects in a Post-Menopausal Osteoporosis Model

**Figure 7:**
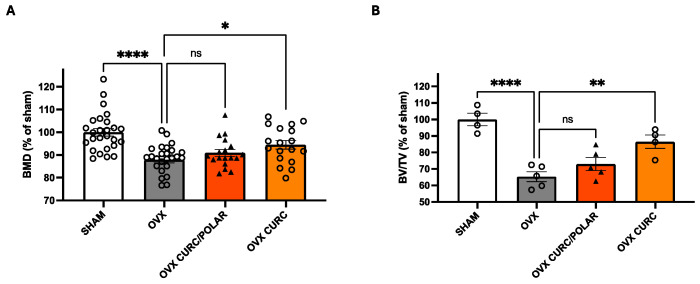
Effect of curcuminoid-containing turmeric extracts on bone loss in OVX rats. Ovariectomized (OVX) rats were treated daily with turmeric extracts (60 mg/kg curcuminoids) vs. vehicle beginning on the day of surgery and followed for 2 months to determine extract effects on OVX-induced bone loss (vehicle-treated OVX vs. sham controls). (**A**) Bone mineral density (BMD) of the trabeculae-enriched distal 25% of the femur, determined by DXA, is reported (n = 18–26). (**B**) Specific treatment effect on trabecular bone loss were determined by micro-CT analysis of the trabecular bone compartment (trabecular bone volume per total volume [BV/TV]) of the distal femur (n = 4/group). Differences between treatments were determined by one-way ANOVA. * *p* < 0.05; ** *p* < 0.01, **** *p* < 0.0001. ns = not significant.

Lastly, given its wide significance for our aging population, effects of the two curcuminoid-only extracts with the best safety profiles (CURC, CURC/POLAR) were compared in a standard model of post-menopausal osteoporosis where bone loss, as in the other models, is Oc driven, albeit mechanistically distinct due to systemic changes in ambient levels of reproductive hormone (e.g., loss of estrogen) [54]. Here, in contradistinction to the tendency for more complex extracts to yield greater benefits in preventing bone destruction in focal inflammation- or cancer-driven models of bone loss, the curcuminoid-only extract (CURC) similar in composition to most commercially available products in the US yielded a significant benefit in preventing bone loss, as determined by bone mineral density (Figure 7A) or microarchitectural examination of the trabecular bone compartment (Figure 7B), while the more complex CURC/POLAR extract was without effect.

### 3.5. Disease-Independent, Host Variables Impacting Turmeric Metabolite Bone Protection

**Figure 8:**
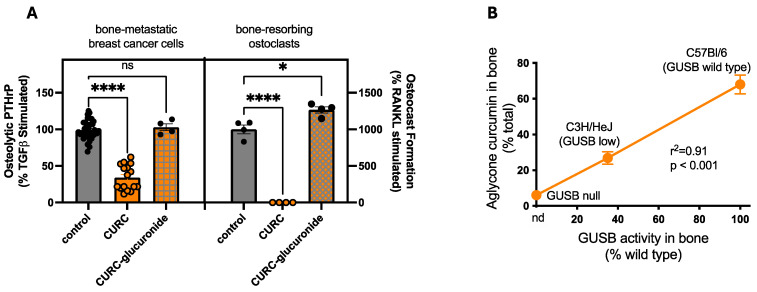
Bioactivity and bone-specific deconjugation of curcumin-glucuronide. (**A**) Comparison of CURC vs. curcumin-glucuronide (CURC-glucuronide) effects on TGFβ-stimulated secretion of PTHrP from human MBD-MB-231 breast cancer cells (left) (n = 4–16/group), or RANKL-stimulated osteoclast formation by RAW 264.7 osteoclast precursors cells (right) (n = 4/group). Differences were determined by one-way ANOVA. * *p* < 0.05; **** *p* < 0.0001. ns = not significant. (**B**) Curcumin metabolite levels in bone marrow isolated from CURC-treated mice are compared for C57BL/6 mice expressing wild type β-glucuronidase (GUSB); GUSB-low C3H/HeJ mice, or GUSB-knock out (GUSB^−/−^) mice. Mice were treated with 100 mg/kg IP CURC or 500 mg/kg oral CURC, which yield similar circulating curcumin metabolite levels, 97–99% of which are glucuronidated [40]. Aglycone (unconjugated) curcumin levels within bone marrow, reported as % of total curcuminoids (curcumin + glucuronidated curcumin), are compared to bone marrow GUSB activity (reported as % of wild type C57BL/6 mice) assayed in a separate set of mice (n = 3–5/group). Association was analyzed by linear regression.

Possible host-specific effects driving in vivo bone responses to turmeric metabolites were also examined. Specifically, since curcuminoids are glucuronidated by the liver upon ingestion and primarily circulate in vivo as glucuronides [40], bioactivity of CURC vs. CURC-glucuronide when targeting cellular responses relevant to bone resorption were assessed. Curcumin-glucuronides, in contrast to inhibitory effects of CURC, had no effect on PTHrP secretion from human breast cancer cells (Figure 8A, left panel) or on RANK-L-stimulated Oc formation (Figure 8A, right panel). Importantly, however, while inactive CURC glucuronides are the primary metabolite perfusing mouse bone following oral (or IP) CURC treatment [40], β-glucuronidase (GUSB), a deconjugating enzyme present at high levels within the bone microenvironment, was able to reverse this process in situ, such that local bioactive CURC levels in bone correlated with local GUSB enzyme activity across mouse strains with variable GUSB expression (Figure 8B).

## 4. Discussion

Ethnobotanical use of medicinal plants has directed modern drug discovery, whose usual goal is to identify a single active principle [55], with isolation of morphine from opium (*Papaver somniferum* L.) for analgesia being a classic early example [56,57]. However, it is now understood that opium-derived alkaloids, such as the smooth muscle relaxant papaverine, can also contribute to pain-relieving properties of traditional opium preparations in certain disease contexts [58]. Thus, any modern, drug-like use of an isolated plant secondary metabolite likely overlooks certain benefits (as well as potential risks) that may accrue from the use of more traditional, complex plant formulations. At the same time, modern pharmacokinetic and pharmacodynamic concepts can readily explain additive, synergistic, and/or antagonistic pharmacodynamic effects of bioactive plant-derived principles when used in combination in a particular health or disease setting [59].

Findings from the studies presented here demonstrate that these same principles apply to traditional vs. modern use of turmeric. In use since 2100 BC, traditional turmeric preparations are clearly chemically complex, contrasting with modern use, where turmeric-derived polyphenols are a focus, as reflected by the curcuminoid-only content of most commercial turmeric dietary supplements in the United States [6]. However, the studies presented here uniquely demonstrate (vs. prior bone-focused studies in which curcuminoid-only extracts were solely studied [2,4,5,21]) that each of turmeric’s secondary metabolites can affect disease outcomes in bone resorptive diseases, with net effects dependent on the mechanistic basis of the bone resorptive disease, which can also be complex and multifactorial.

Comparison of the isolated or combined pharmacodynamic effects of turmeric’s two classes of secondary metabolites across resorptive bone diseases with distinct mechanistic drivers yielded intriguing results. For example, purified curcuminoid were the least effective in blocking bone resorption driven by either adjacent focal inflammation (rheumatoid arthritis model) or adjacent tumors (breast cancer bone metastases model). Instead, and consistent with possible synergistic pharmacodynamic effects in vivo, CURC (with greater effect in combination with polysaccharides) or TEO each blocked bone loss in the arthritis model when administered in isolation. In vitro studies provided evidence of possible multifactorial targets for each metabolite, some of which were disease specific. CURC and TEO each blocked RANKL-mediated osteoclast formation, albeit by possibly different mechanisms since only CURC blocked RANKL-stimulated NF-κB. In contrast, CURC vs. TEO effects on PTHrP secretion, which drives bone loss in both the arthritis and breast cancer models, varied by cell type (human synoviocyte vs. breast cancer cells) and/or stimulus (IL-1β vs. TGFβ), as both blocked IL-1ß stimulated PTHrP secretion from synoviocytes while only CURC-containing extracts blocked TGFβ-stimulated PTHrP secretion from breast cancer cells. While not examined in this study, evidence indicates that oxidized curcumin metabolites may mediate some of curcumin’s biological effects [60], such as inhibiting the activation of NF-kB, a key mediator of inflammation [61], via formation of a covalent protein adducts [62]. Thus, potential distinct biological effects of CURC versus TEOs may be dictated, in part, by their bioactive metabolites.

The benefits of CURC-only, but not CURC/POLAR extracts, in preventing bone loss in the post-menopausal osteoporosis model clearly diverged from benefits documented for complex extracts in preventing inflammation- and cancer-induced bone loss. Distinct underlying mechanisms may explain these differences, as for example, systemic bone loss in the OVX model occurs secondary to changes in reproductive hormones (which polysaccharides could, theoretically, further antagonize), while prevention of focal bone loss by turmeric extracts in the arthritis model clearly tracked with suppression of adjacent joint inflammation. Findings across all three bone models, however, clearly demonstrated that CURC-only preparations that are significantly similar to those sold commercially, while not necessarily the most potent product, did significantly prevent bone loss in every model, with an increase in adverse effects when TEO and/or polar compounds were present, possibility limiting clinical utility.

While bone resorption was the primary focus of these comparative studies, also of clinical interest were metabolite effects on inflammation-driven vascular endothelial activation and elevated CRP levels in the RA model, both of which are risk factors for cardiovascular (CV) disease [46,63], which occurs more frequently in individuals with RA [64]. Interestingly, only TEO reduced CRP levels in the RA model, while CURC was the sole metabolite preventing cytokine-mediated NF-κB activation and ICAM induction in vascular endothelial cells, suggesting possible benefits in CV-risk reduction using turmeric metabolites in combination. However, the precise clinical significance of these findings is not clear since (1) oral (vs. IP) TEO treatment in the RA model may be less efficacious in reducing inflammation [11] and (2) CRP elevations, while they are used clinically to follow RA disease activity in humans [65], are less dramatically altered in rodent models of inflammation [47].

Extract-specific inhibition of granuloma formation in the inflammation model, which is also a PTHrP-dependent process [35], was also of potential clinical relevance. While inhibitory effects of CURC vs. TEO in blocking PTHrP release from rheumatoid synoviocytes were similar in vitro, only TEO-containing turmeric extracts, or CURC extracts containing polysaccharides, inhibited granuloma formation in vivo, possibly suggestive of mechanism(s) unrelated to PTHrP blockade. In any case, because granuloma formation can be a beneficial host response with certain chronic infections [45], CURC-only may have a better safety profile in these settings due to its lack of effect on host granulomatous responses.

One limitation of these studies is the use of female rodents in all models to study turmeric metabolite bone effects. However, this design was driven by the significant clinical burden of these diseases in women, who are also more frequent dietary supplement users [19], and, interestingly, also appear to have greater curcuminoid bioavailability, as compared to men [66,67].

In summary, these studies suggest that additive bone protective effects of multiple turmeric metabolites are possible and can be mediated by curcuminoids, terpenes, and/or polysaccharides. Net bone effects of any complex turmeric product are likely attributable to the multiplicity of pharmacodynamic targets for a given metabolite, as well as interacting effects of different metabolites, both of which are disease specific. Although it is not addressed here but is relevant for clinical use, the effects of chemically complex turmeric products in the clinical setting may also be altered by pharmacokinetic metabolite interactions, as terpenes and polysaccharides can both be harnessed to alter curcuminoid bioavailability. Lastly, because curcuminoids, like many other dietary polyphenols, circulate in vivo as inactive glucuronidated “pro-drugs” [68], medicinal effects of this secondary turmeric metabolite are also likely host-dependent, requiring enzymatic bioactivation by β-glucuronidase (GUSB) [40], which interestingly is present at very high levels in bone [40] and in RA (vs. normal) joints [69], and, conversely, whose absence is associated with increased joint inflammation [70]. Emerging evidence also suggests that GUSB activity may correlate with musculoskeletal health in post-menopausal breast cancer survivors [71], a population that, as with RA sufferers, suffers from accelerated frailty [72,73] and also reports a high prevalence of turmeric use [20]. While ancient in its usage, our understanding of the underpinnings of turmeric’s medicinal effects, and thus its potential benefits for modern use, in some ways remains in its infancy.

## 5. Conclusions

Turmeric’s bone protective effects are not limited to the curcuminoids, with both secondary metabolites, and also rhizome-derived polysaccharides, having additional and/or additive bone protective effects that appeared disease- and cell target-specific. Thus, it is possible that some medicinal bone benefits of traditional turmeric preparations may be lost with modern usage, which is curcuminoid-focused. However, it is notable that curcuminoid-only products analogous to many commercial turmeric dietary supplements, while not always the most potent in vivo and also lacking in biological effect for certain cellular targets, still offered significant bone protection across all bone disease models. For evidence-based clinical use, additional carefully designed clinical trials will be required.

## Figures and Tables

**Table 1:** In vivo models.

Disease Being Modeled	In Vivo Model	Animal	In Vivo Turmeric Treatments			
			CURC	CURC/POLAR	TEO	COMPLETE
Arthritis, rheumatoid	SCW-induced arthritis	rat, female	X	X	X	X
Bone tumor, osteolytic breast cancer	Bone-disseminated human breast cancer cells	mouse, female	X	X		X
Osteoporosis, post-menopausal	OVX-induced bone loss	rat, female	X	X		
(bone GUSB levels, variable)	various mouse strains	mouse, female/male	X			

CURC, curcuminoids-only; CURC/POLAR, curcuminoids plus polar compounds; TEO, turmeric essential oil; COMPLETE, contains curcuminoids, turmeric essential oils and polar compounds; SCW, streptococcal cell wall; ovariectomy, OVX; β-glucuronidase, GUSB.

## Data Availability

The data presented in this study, which are a compilation of findings across multiple projects, are available on request from the corresponding author.
